# Gene expression variation and parental allele inheritance in a *Xiphophorus* interspecies hybridization model

**DOI:** 10.1371/journal.pgen.1007875

**Published:** 2018-12-26

**Authors:** Yuan Lu, Mikki Boswell, William Boswell, Susanne Kneitz, Barbara Klotz, Markita Savage, Raquel Salinas, Rebecca Marks, Janine Regneri, John Postlethwait, Wesley C. Warren, Manfred Schartl, Ronald Walter

**Affiliations:** 1 The *Xiphophorus* Genetic Stock Center, Department of Chemistry and Biochemistry, Texas State University, San Marcos, Texas, United States of America; 2 Physiological Chemistry, Biozentrum, University of Würzburg, Würzburg, Germany; 3 Comprehensive Cancer Center Mainfranken, University Clinic Würzburg, Würzburg, Germany; 4 Institute of Neuroscience, University of Oregon, Eugene, Oregon, United States of America; 5 Bond Life Science Center, University of Missouri, Columbia, Missouri, United States of America; 6 Hagler Institute for Advanced Studies and Department of Biology, Texas A&M University, College Station, United States of America; Auburn University, UNITED STATES

## Abstract

Understanding the genetic mechanisms underlying segregation of phenotypic variation through successive generations is important for understanding physiological changes and disease risk. Tracing the etiology of variation in gene expression enables identification of genetic interactions, and may uncover molecular mechanisms leading to the phenotypic expression of a trait, especially when utilizing model organisms that have well-defined genetic lineages. There are a plethora of studies that describe relationships between gene expression and genotype, however, the idea that global variations in gene expression are also controlled by genotype remains novel. Despite the identification of loci that control gene expression variation, the global understanding of how genome constitution affects trait variability is unknown. To study this question, we utilized *Xiphophorus* fish of different, but tractable genetic backgrounds (inbred, F_1_ interspecies hybrids, and backcross hybrid progeny), and measured each individual’s gene expression concurrent with the degrees of inter-individual expression variation. We found, (a) F_1_ interspecies hybrids exhibited less variability than inbred animals, indicting gene expression variation is not affected by the fraction of heterozygous loci within an individual genome, and (b), that mixing genotypes in backcross populations led to higher levels of gene expression variability, supporting the idea that expression variability is caused by heterogeneity of genotypes of cis or trans loci. In conclusion, heterogeneity of genotype, introduced by inheritance of different alleles, accounts for the largest effects on global phenotypical variability.

## Author summary

Phenotypical variability is a multi-factorial phenomenon. Although it has been shown that inheriting certain gene is associated with lower phenotypical variability, how genome complexity affect phenotypical variability is still unclear. To study this question, we used inbred *Xiphophorus* fish, backcross interspecies hybrids, and F_1_ interspecies hybrids between select *Xiphophorus* species to model genetic composition with minimum, medium, and maximum heterozygosity respectively, and measured their global gene expression variability. We found gene expression variation is not affected by the percentage of heterozygous loci in individual genome, but instead related to heterogeneity of genotype at local or remote loci.

## Introduction

Gene expression is regulated by complex genetic interactions [1–5]. The quantitative nature of gene expression provides a measurable trait. In human populations, Genome Wide Association Studies (GWAS) have revealed a plethora of genomic hotspots that are associated with different traits, diseases and are responsive to environmental stimuli [1]. Such studies have also been applied to model organisms utilized in medical research, or for trait selection in commercial applications [6–8]. Since the early 2000’s, when microarray and sequencing based transcriptome profiling became widely used to measure global gene expression, it has been known that gene expression variation broadly exists. Transcriptional variation was found to be environment, sex, and age related [9–11], and recently it has also been determined that genetic variants play a role in the variation of gene expression [12]. Despite efforts in identifying loci associated with gene expression variation, understanding of the source of transcriptional variability is still superficial [12–14].

An earlier study showed genetic variants contribute to expression variation at a particular locus, suggesting that inheritance of specific alleles may change gene expression variation [12]. This is true for certain genes, however, it is not known how the genome composition may affect trait variations globally. For example, does a population with higher degree of heterozygosity exhibit a larger range of inter-individual gene expression variation? Herein, we seek to understand what accounts for within-population gene expression variation at the global level. Outbred population data are not suitable to answer this question because these genomes are highly heterogeneous and multiple alleles for each gene within a population may confound interpretation of results obtained. In addition, a homozygous genome is required as a control to estimate variations in basal gene expression. Therefore, we adopted a unique animal model system, namely fish lines of the genus *Xiphophorus*, to test the hypothesis that genome state, in regard to polymorphic complexity, affects gene expression variation. *Xiphophorus* is a tropical freshwater fish genus consisting of 26 known species. Their aquatic habitats range from northern Mexico southward along the Sierra Madre uplift into Guatemala, Belize and Honduras. The taxa are clustered into three common groups: Northern Swordtails, Southern Swordtails, and Platyfish. A valuable and unique feature of *Xiphophorus* is that hybridization of different *Xiphophorus* species produces viable and fertile interspecies hybrid offspring. This feature allows the study of genetic interactions from two genomes that have been separated 6–8 million years of evolution (For review, see [15]). *Xiphophorus* interspecies backcrosses have served as a longstanding model for the genetics underlying cancer development and were utilized to propose the existence of tumor promoting (oncogenes) and tumor suppressor genes [15]. Interspecies hybridization leads to several phenotypical changes, such as pigmentation enhancement and development of melanoma in certain *Xiphophorus* hybrids (For review: see [15]). More importantly, this hybridization model allows us to track allelic expression in the parental species, F_1_ interspecies hybrid, and backcross hybrid progeny, enabling quantification of both genomic composition and allele expression on a locus-by-locus basis. Allele segregation through recombination in backcross progeny can also be used to estimate higher orders of gene expression regulation. The genomes of different *Xiphophorus* species are quite divergent, allowing high-resolution SNP mapping within interspecies hybrids. SNPs on average appear every 80 bp between *X*. *maculatus and X*. *hellerii* species [16], compared to 1 in 13.4 kbp among inbred mouse strains [17] and 1 in 1,200 bp in humans of different populations [18]. The availability of high quality genome assemblies in key *Xiphophorus* species (i.e., *X*. *maculatus*, *X*. *hellerii*, and *X*. *couchianus*) allows facile identification of genetic differences among this genus, and enables study of the interaction between two parental alleles within the genome of *Xiphophorus* interspecies hybrids [16,19–21].

Herein, we report results of a retrospective study that re-analyzes a previously established dataset utilizing gene expression as a quantifiable trait among individual *Xiphophorus* having different, but known genetic backgrounds (i.e., inbred *X*. *maculatus* and *X*. *couchianus* parental species, F_1_ interspecies hybrids between *X*. *maculatus* and *X*. *couchianus*, and different generations of backcross hybrids between the *X*. *maculatus* and *X*. *couchianus* or between *X*. *maculatus* and *X*. *hellerii*). This experimental design served to model genetic backgrounds with minimum, maximum and medium heterozygosity, respectively, and to allowed us to measure global transcriptional trait variability. We report estimates of the relationship between gene expression variance, and genome complexity, within different populations and interspecies crosses.

## Results

### Gene expression variability within *Xiphophorus* inbred, hybrid and human populations

**Fig 1:**
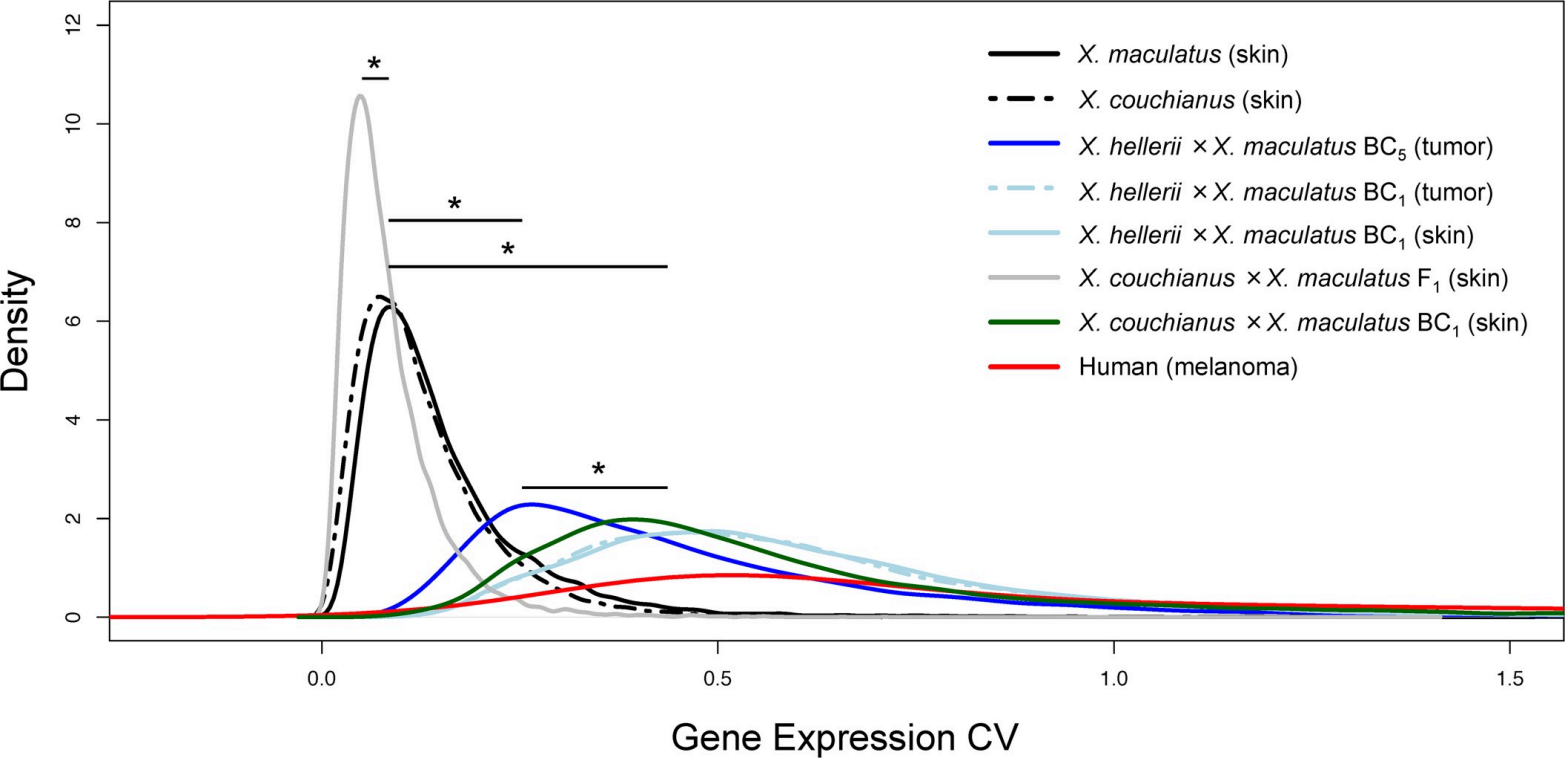
Coefficient of Variance Density in inbred *X*. *maculatus*, melanoma-bearing *Xiphophorus* interspecies hybrids and human melanoma. Gene expression profiles of skin from inbred *X*. *maculatus*, *X*. *couchianus*, F_1_ *Sp-couch* hybrids, *X*. *hellerii* × (*X*. *maculatus* × *X*. *hellerii*) BC_1_ interspecies hybrids, *X*. *couchianus* × (*X*. *couchianus* × *X*. *hellerii*) BC_1_ interspecies hybrids, melanoma samples from *X*. *hellerii* × (*X*. *maculatus* × *X*. *hellerii*) BC_1_ and BC_5_ interspecies hybrids, and human melanoma were used to calculate gene expression coefficient of variance among each population. A density plot was used to represent the distribution of coefficient of variation. Kolmogorov–Smirnov test was performed to test whether the distribution of CV are statistically different between different genetic backgrounds (BC_1_ vs. BC_5_ expression CV values p-value < 2.2E-16; BC_1_ vs. inbred expression CV values p-value < 2.2E-16; F_1_ vs. inbred expression CV values p-value < 2.2E-16). * means a particular test led to statistically significant difference.

To estimate overall gene expression variation, coefficient of variation (CV) of all expressed genes was calculated in inbred *X*. *maculatus* and *X*. *couchianus* (skin), *X*. *hellerii* × (*X*. *maculatus* × *X*. *hellerii*) BC_5_ hybrid (melanoma), *X*. *hellerii* × (*X*. *maculatus* × *X*. *hellerii*) BC_1_ hybrid (skin and melanoma), *X*. *couchianus* × (*X*. *maculatus* × *X*. *couchianus*) BC_1_ hybrid (skin), and *X*. *maculatus* × *X*. *couchianus* F_1_ hybrid (skin). As a comparison, gene expression CV for each locus of human melanoma patient populations was also calculated. Inbred *X*. *maculatus* and *X*. *couchianus* exhibited gene expression CVs centered around 0.087, and 0.074 respectively (Fig 1). Melanoma samples from *X*. *hellerii* × (*X*. *maculatus* × *X*. *hellerii*) BC_5_ interspecies hybrids, in which by theory 98.4% of their genome is derived from the *X*. *hellerii* parent, showed a gene expression CV distribution centered around 0.264 (Fig 1). Both skin and melanoma tumor samples from *X*. *hellerii* × (*X*. *maculatus* × *X*. *hellerii*) BC_1_ showed similar distribution of gene expression CVs, with skin samples showing a CV distribution centered around 0.493, and tumors showing CV distributions centered around 0.459 (Fig 1). *X*. *couchianus* × (*X*. *maculatus* × *X*. *couchianus*) BC_1_ hybrid exhibited a slightly left-shifted CV distribution compared to *X*. *hellerii* × (*X*. *maculatus* × *X*. *hellerii*) hybrids, with a gene expression CV distribution centered around 0.393. As a comparison, gene expression profiles from TCGA human melanoma datasets showed a CV value mostly enriched around 0.54. Additionally, human gene expression showed a long trailing tail on the CV distribution compared to that of parental or backcross hybrids (Fig 1). Unexpectedly, F_1_ interspecies hybrids, which had inherited alleles from two inbred species segregated at a 1:1 ratio, showed a CV distribution shifted left, toward lower values compared to inbred animals (Fig 1). Most genes in the F_1_ hybrids showed expression CV’s of 0.047 (Fig 1). The CV values distributions of F_1_, BC_1_ and BC_5_ were all statistically different from the distribution of inbred animal (Fig 1).

We identified 318 genes that showed less variability (p-value<0.05, CV_F1_/CV_parental_ < 0.25), and 57 genes showed more variability (p-value<0.05, CV_F1_/CV_parental_ > 4) in the F_1_ compared to the *X*. *maculatus* parent. In comparison, 568 genes showed less variability (p-value<0.05, CV_F1_/CV_parental_ < 0.25), and 74 genes showed more variability (p<0.05, CV_F1_/CV_parental_ > 4) in the F_1_ compared to *X*. *couchianus*. The observation of more genes exhibiting less variability in F_1_, regardless of which parental species ones compare to, is consistent with the left shift of CV distribution curve for F_1_’s.

We also assessed the allelic contribution to overall gene expression in the F_1_ interspecies hybrids, and assessed allele specific expression variability. Genes that showed dominant expression by one parental allele [i.e, One allele contributes more than 80% of the gene expression, or Log_2_(*X*. *maculatus* allele expression / *X*. *couchianus* allele expression) > 2 or <-2] exhibit a smaller degree of expression variability for the dominantly expressed allele (S2 Fig).

### Heterogeneity of genotype accounts for gene expression variance

**Fig 2:**
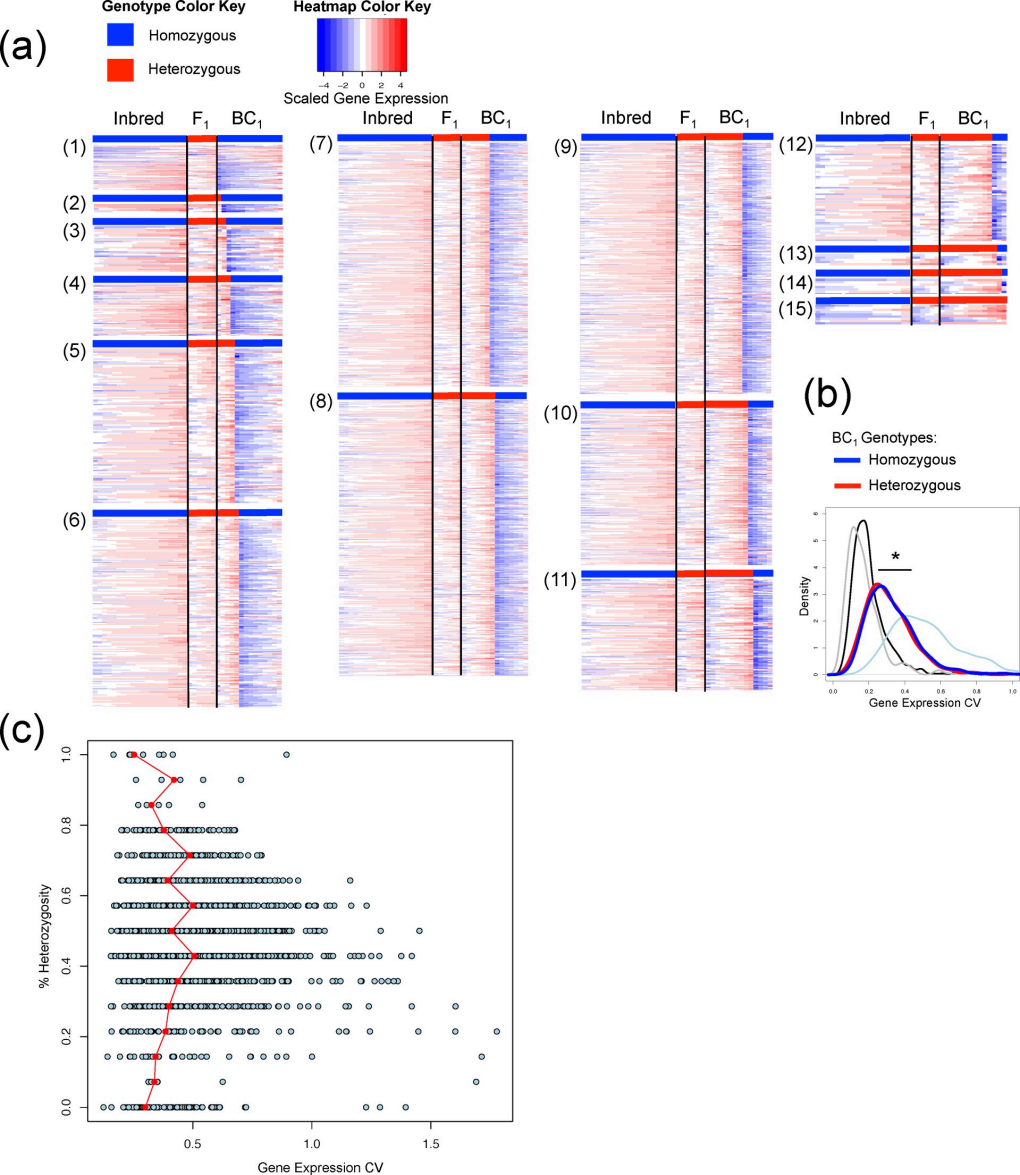
Heterogeneity of genotypes increases gene expression variance. Gene expression variance analyses were performed on all genotyped genes from BC_1_ animals. **(a)** Heatmap showing normalized and scaled gene expression in inbred, F_1_ and BC_1_ genetic backgrounds. Expression values of a particular gene in different samples were plotted in ascending order. Heatmaps were separated into different blocks with each representing different numbers of heterozygous/homozygous individuals of BC_1_ animals. Blue bars on top of each heatmap block highlighted homozygous samples, red bars highlighted heterozygous samples. **(b)** Gene expression CV distribution of inbred parental (black line), F_1_ (gray line), all BC_1_ (light blue line) individuals. CV distributions were recalculated for genes that are homozygous in 50% of BC_1_ individuals (blue line), and heterozygous in the other 50% progeny (red line). The segregation of genes of different genotypes decreased the CV values. * means a particular test led to statistically significant difference. **(c)** Expression CVs were calculated for genes that show different genotypes within BC_1_ individuals. X-axis coordinate of blue dots showed CV value of a gene, and X-axis coordinate of red dot showed CV value where most CV values were observed. When there is no heterogeneity of genotypes (top and bottom rows), CV values were minimum. In contrast, when there was a mixture of different genotypes, inter-individual gene expression CV values increased.

Although F_1_ interspecies hybrids had the highest percentage of genome heterozygosity in terms of ancestry allele inheritance, there was neither a shift of CV toward larger values, nor a widened distribution of gene expression variance as would be expected if variance is directly related to the percentage of genome heterozygosity (Fig 1). Inbred and F_1_ animals are similar in that they have homogeneous genotypes at almost all loci for all individuals in their respected populations. In contrast, BC_1_ animals have heterogeneous genotypes with respect to ancestry at each locus represented throughout the population. To test the hypothesis that heterogeneity of ancestry accounts for gene expression variance, we compared the expression of genes in all BC_1_ samples to those of the inbred and F_1_. A total of 2,073 genes can be genotyped in all BC_1_ animals (Fig 2). Gene expression among BC_1_ animals exhibits a large variation. A differential expression test showed that among the 1,944 genes shown to be heterozygous for *X*. *maculatus* and *X*. *hellerii* alleles, or homozygous for *X*. *hellerii* alleles in at least 3 of the 14 BC_1_ individuals, 1,387 of them presented differential expression (FDR adjusted p-value <0.05) between individuals of different genotypes of ancestry (Fig 2A; S2 Table). We independently calculated the expression CV distribution of genes that are heterozygous and homozygous (Fig 2B). Expression CV’s of genes of the same genotype showed the same distribution, and the CV density curve of both genotype groups shows a left-shifted distribution pattern toward the inbred and F_1_ animals. This observation suggests the genotype of a locus majorly accounts for the locus-specific expression variation.

Gene expression CV density analysis was performed on genes showing different heterozygous ratios within the BC_1_ animal population. Genes that showed exclusively the same genotype among BC_1_ individuals [i.e., all homozygous, or all heterozygous, Fig 2A (1), (15)] also exhibited the smallest CV values (Fig 2C top and bottom rows), while genes that showed mixed inter-individual genotypes exhibited larger expression CV values (Fig 2B and 2C).

### Identification of genetic interactions in interspecies *Xiphophorus* hybrids

**Fig 3:**
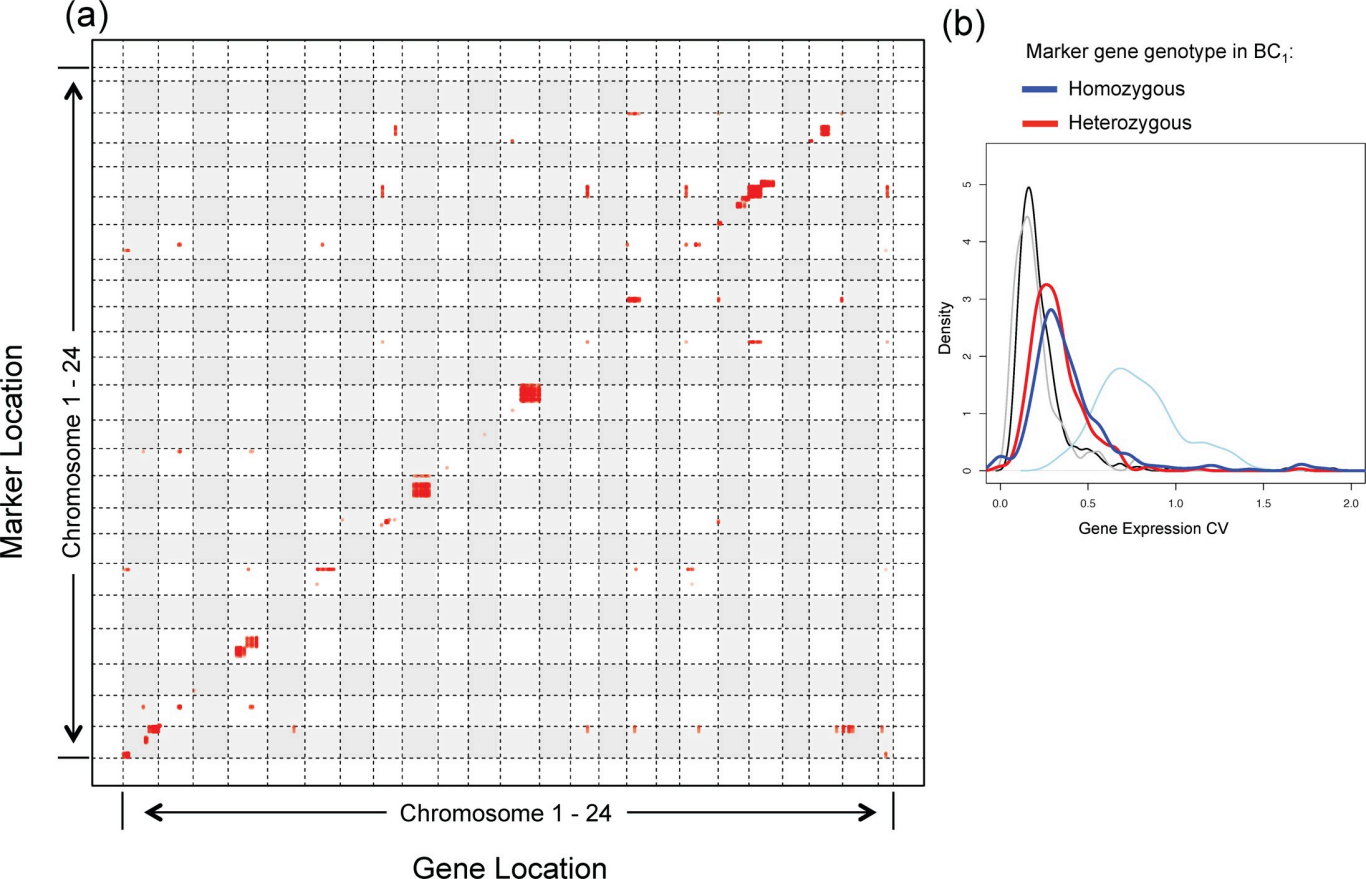
Association between gene expression and genome-wide genotype. Allele specific and overall gene expression were performed in 14 melanoma-bearing *Xiphophorus* BC_1_ interspecies hybrid progeny. Allele specific gene expressions were used to infer genotype of each individual. Association between genotype and overall gene expression were performed. A statistical significant interaction was determined by FDR adjusted p-value < 0.05. **(a)** A total of 7511 pairs of genetic interactions that involved 434 variants and expression of 327 genes were found to be significant. In the plot, each horizontal and vertical strip represents one chromosome. Widths of the strips represent the physical length of each chromosome. Gene and genetic marker physical locations were plotted on X- and Y- axis respectively. Red dots the genotype (heterozygous for *X*. *maculatus* and *X*. *hellerii* alleles, or homozygous for *X*. *hellerii* allele) of a gene is associated with the higher or lower expression of another gene. In cases where genotypes of several genes that are adjacent to each other regulate the expression of the same gene, red dots appears to form a vertical line. **(b)** Expression of 289 genes is associated with cis-variants and expression of 111 genes is associated with trans-variants. CV distribution is recalculated for the 289 and the 111 genes based on marker genes (i.e., variants) genotypes. Gene expression CV distribution of inbred parental (black line), F_1_ (gray line), all BC_1_ (light blue line) individuals. CV distributions were recalculated when 50% of BC_1_ individuals were homozygous for the associating locus/loci (blue line), and the other 50% were heterozygous for associating locus/loci (red line). The left shift of the dark blue curve and red curve means separating genes of different genotypes decreased the CV values.

It is well known that gene expression can be modulated by both local and remote regulatory sequences [5,22]. These genetic interactions, along with the inter-individual genotype differences (i.e., genetic background) of both regulatory genes, and genes-to-be-regulated, may add more complexity to gene expression variance. To identify such interactions, we tested whether the genotype (i.e., homozygous for the *X*. *hellerii* allele, or heterozygous for the *X*. *maculatus* and *X*. *hellerii* alleles) of a gene, which we designated as marker gene, correlated to the higher/lower expression of other genes within the *Xiphophorus* genome. A genotype-gene expression association analyses identified expression of 327 genes that appear affected by 434 marker genes (FDR adjusted p-value <0.05; Fig 3A; S1 Table). We determined that if the genotype of marker gene A was associated with the expression of gene B, and marker gene A is located on the same chromosome as the gene B, marker gene A regulates gene B in a *cis*-manner. Alternatively, if marker gene A is located on different chromosome from gene B, marker gene A regulates gene B in a *trans*-manner. 289 of the 327 genes were found regulated by cis-variants, while 111 genes were regulated by trans-variants, and 73 genes were regulated by both cis- and trans-variants. (Fig 3A; S1 Table). Among these genes, 321 of the 327 genes showed differential expression between individuals that are homozygous and heterozygous for the associated genetic variants (i.e., marker genes; FDR adjusted p-value < 0.05). Similarly, as we observed earlier in this study, gene expression CV pattern exhibits a left-shift if individuals with heterozygous or homozygous associating genes marker are calculated separately, compared to all individuals were calculated together (Fig 3B; S3 Fig). This suggests stratifying the CV by genotypes serves to reduce gene expression variance (i.e., CV).

## Discussion

The genetic contribution to phenotype expression is of long-standing research interest. GWAS, especially eQTL studies, have revealed a plethora of local or remote genetic interaction pairs (For review: see [1]). These studies focus on identifying loci (i.e., QTL) with a genotype associated with the mean expression of another genes (i.e., eGene). It has also been recently forwarded that gene expression variability is genetically controlled. For example, inter-cellular gene expression variability analyses on four-cell, eight-cell, morula and blastocytes stages revealed gene expression variability changed during early embryo development [23]. Also, higher transcriptional variability is associated with Schizophrenia and an aggressive subtype of chronic lymphocytic leukemia, suggesting that loss of delicate transcriptional regulation is associated with disease [24,25]. Like eQTL-regulated genes, expression variability may also be controlled by particular loci [12]. Despite these discoveries, how genome complexity contributes to overall gene expression variation is not understood.

**Fig 4:**
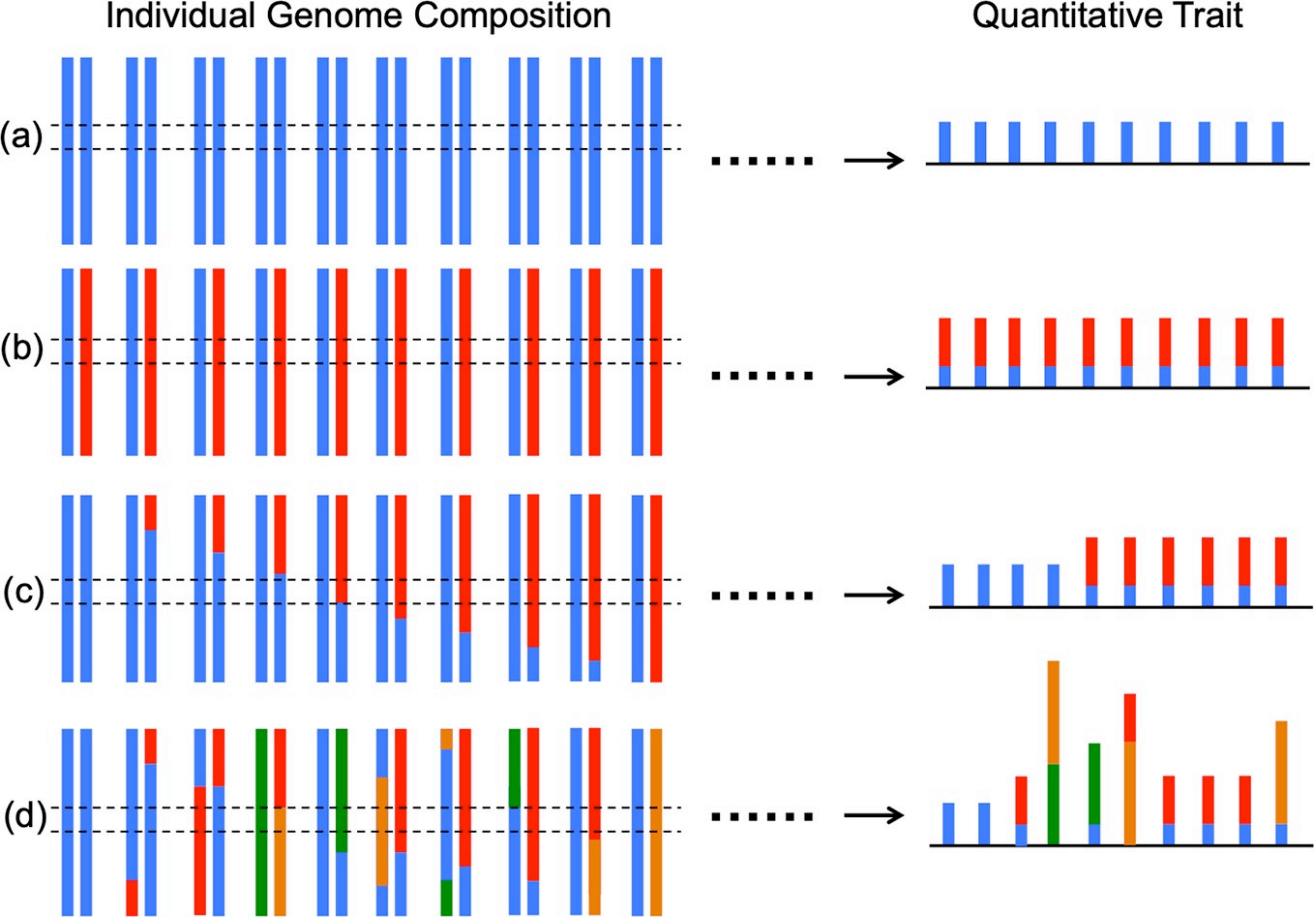
Heterogeneity of genotype is associated with quantitative trait variation. This figure summarizes how genome composition effect phenotype expression and how heterogeneity of genotype related to inter-individual trait variance. On the left side of the figure, each pair of bars represents one pair of chromosome, with each color represented an allele. On the right side, the trait controlled by the region between the dashed lines is presented for each individual of a population. Populations (a) and (b) have no heterogeneity of genotype for all individuals. Although their percentages of heterozygous loci are different, the trait controlled by each locus show the least amount of variation among each population. Recombination introduces heterogeneity of genotype in (c) controlled population and (d) wild population. The combination of varied genotypes, and different alleles of a locus within a population accounts for the inter-individual trait variation.

Our study utilized gene expression as a quantifiable trait to address the question of how genome complexity affects trait variation. To do this we compare the inter-individual gene expression variability of different genetic backgrounds. The control inbred *Xiphophorus* species used in this study (i.e., *X*. *maculatus* and *X*. *couchianus*) has been maintained by line-breeding (i.e., brother-sister matings). *X*. *maculatus* has been maintained this way for over a hundred generations, and *X*. *couchianus* for over 85 generations. *X*. *hellerii* have been maintained by intercrossing two lines that exhibit sword tail color variation for over 60 generations in the *Xiphophorus* Genetic Stock Center (San Marcos, TX, USA). The measured gene expression variation of inbred laboratory species, and in particular for *X*. *maculatus*, is expected to be minimal because all inbred individuals are homozygous and should have inherited the same set of alleles in different individuals. From a gene expression regulation standpoint, gene expression is under the regulation of a transcription factor that selectively binds to a regulatory sequence [e.g., promoter; for review, see [26]. This genetic interaction is not expected be affected by different alleles of the transcription factor, or the gene under regulation, within an inbred genome (i.e., genetically homogeneous). In contrast, in a backcross hybrid genome, where genomic information is provided by two divergent species, polymorphisms of both the transcription factor, and the regulatory binding site of the transcription factor in gene targets, may be affected, and such genetic interactions could influence gene expression. Additionally, each backcross individual may inherit different modes of genetic interaction (i.e., heterogeneous). The observed increase of gene expression variation, in concert with increased fractions of heterozygous loci in BC_5_ and BC_1_ animals, is a reflection of such an allelic effect on gene expression regulation. The *Xiphophorus* interspecies hybrids are expected to have two alleles, each inherited from one parental species, for each gene within the population. This is not the case for human populations, which may contain many different alleles at each locus within the population. It is suggested that higher numbers of allelic variants in humans may contribute to the thick tailing of the gene expression variation density curve (Fig 1; Fig 4). Considering the data collected from inbred and different generations of backcross animals, the inter-individual genome complexity appears to be associated with gene expression variation [12,27].

However, considering that F_1_ animals have the largest percentage of heterozygous loci, and the potential to express different parental alleles in different individuals, it was surprising to observe that F_1_ interspecies hybrids show a smaller level of gene expression variation (i.e., CVs) than inbred animals (Fig 1). In this case, the left-shift of the CV distribution curve of F_1_ gene expression is not due to the lower sample size of F_1_ animals. This is because when we randomly selected 6 inbred animals and calculated their gene expression CV distributions, the sample size-matched inbred samples also show CV density curve peaks located at higher values compared to the F_1_ samples (S1 Fig). It is well known that sequencing read counts of biological replicates in gene expression profiling tasks (i.e., RNA-Seq or microarray) follow a trend that highly expressed genes exhibit less variability than the lowly expressed genes [28–31]. Therefore it is also worth to note that this statistical effect does not account for the above observation. Because if this observation is due to statistical effect, one may expect the expression of a majority of genes would show higher expression (i.e., up-regulation) than either parental species. However, this is not true in earlier studies on the same *Xiphophorus* interspecies hybrids, and amazon molly, a species originated from an interspecies hybridization event [32,33]. The simplest interpretation of this observation (i.e., F_1_ interspecies hybrid exhibited less gene expression variability than either parental species) is that allelic expression is not random in each individual. Instead, the allele selections of regulatory genes and target genes are rather consistent among all F_1_ individuals from a given interspecies cross (Fig 1, Fig 4). Similar observation that F_1_ hybrids show less variability is not unforeseen. A plethora of different quantifiable traits from many different model systems have shown smaller variation in F_1_ offspring between two strains, than for the inbred parental. This observation was explained by the “homeostasis gene” hypothesis [34]. This hypothesis suggests there are certain “genes” that control genetic homeostasis. Therefore inheriting recessive alleles of a homeostasis-controlling gene may result in larger transcriptional phenotypic variance, compared to the condition of inheriting at least a dominant homeostasis allele (i.e., hybrid). Allelic expression and variability analyses showed that genes exhibiting less variability in F_1_, compared to parental species, are dominantly expressed by the parental alleles that showed less variability in the hybrid genetic background (S2 Fig, gray and black dots). However, higher uniformity in expression of genes that exhibited less variability in hybrids does not seem to be a feature of the parental species (S2 Fig, blue dots). Our data indicate that F_1_ interspecies hybrids may inherit dominant homeostasis gene(s) from one parental species that “stabilize” gene expression, and therefore show a globally more uniform transcriptional phenotype among F_1_ population. We have no evidence that genes with less expression variability in the hybrid background are indeed “homeostasis genes”, but they are likely to be under the control of such regulators. On a separate note, the molecular genetic mechanism of heterosis, especially in vertebrate animals, is still unclear. Although it is beyond the scope of this study to investigate the mechanism of heterosis, and it is not observed that inbred *Xiphophorus* strains exhibit inbreeding depression, it will be an interesting future topic to study in order to assess whether a higher level of phenotypical uniformity of F_1_ interspecies hybrids is related to heterosis [35]. In summary, the expression variation comparisons between inbred and F_1_ interspecies hybrid animals showed that genome heterozygosity alone does not explain inter-individual gene expression variance.

The finding that local or remote genetic variant genotypes are associated with different levels of gene expression supports our hypothesis that a heterogeneous genotype in a population accounts for inter-individual gene expression variance (Fig 2 and Fig 3). The observation that more genes are regulated by cis-variants than trans-variants is consistent with findings from numerous QTL studies that gene expression is majorly regulated by cis-variants [36–38]. Separating animals of different genotypes resulted in lower CV values that are close to populations that do not exhibit genotype heterogeneity (Fig 2, Fig 3 and Fig 4). This suggests that genetic variants of the same genotype affect local and remote gene expression similarly. We expected that calculating gene expression CV distribution of individuals of the same genotype separately would fully change the CV values to a condition similar to the homogeneous genotype (i.e., inbred and F_1_ hybrid). However, this was not observed (Fig 2 and Fig 3) as genes of BC_1_ animals of the same genotype still showed slightly larger CVs than those of inbred or F_1_ hybrids, even after we separately calculated CV distribution, and after stratifying both cis- and trans-variant genotypes (S2 Fig). We hypothesize this is due to gene regulation by multiple loci, with each regulatory locus that leads to a heterogeneity of genotypes. If the expression of a gene is regulated by both cis- and trans-variants, its expression can be very different among individuals harboring different combinations of genotypes in regard to cis- and trans-variants; therefore the expression pattern within a population is more complex compared to genes that are only regulated by cis- or trans-variants. Separately calculating gene expression variability of individuals whose cis- and trans-variant genotypes are the same should completely eliminate the genetic contribution to gene expression variability. Unfortunately, we do not have the luxury of larger sample sizes that are required to test this idea. However, eQTL studies focused on complex traits or diseases have provided evidence to support this hypothesis [39–41]. It is also worth the attention that ages of backcross animals used in this study are higher than the inbred parental animals or F_1_ hybrids. It is known that age is a factor that contributes to gene expression regulation [42–44]. However, the observation that gene expression variability decreased after separating the expression by ancestry suggests that age is not a main driving factor for the increased variability in backcross animals.

Overall, this study has revealed that (1) amplitude of transcriptional phenotype variation is a genetic background-dependent parameter; and (2) inter-individual heterogeneity of genotype accounts for global gene expression variation.

## Materials and methods

### Ethics statement

All animals used in this study were kept in accordance with the protocol approved by the Texas State University Institutional Animal Care and Use Committee (IACUC protocol #2015107711). All BC_5_ fish were kept in accordance with the applicable EU and national German legislation governing animal experimentation as authorized by permit 568/300-1870/13 issued by the Veterinary Office of the District Government of Lower Franconia, Germany, in accordance with the German Animal Protection Law (TierSchG).

### Animal model

*X*. *maculatus* used in this study were supplied by the *Xiphophorus* Genetic Stock Center (XGSC; For information see: http://www.xiphophorus.txstate.edu/). The *X*. *maculatus* Jp 163 B utilized herein have been inbred by brother-sister matings for 104 or 105 generations. All *X*. *maculatus* used in this study are male that are 9–12 months old. The *X*. *couchianus* have been inbred for 85 generations. All inbred animals used in this study are male that are 12 months old.

F_1_ interspecies hybrids between *X*. *maculatus* and *X*. *couchianus*, designated Sp*-couch*), were produced in the XGSC by crossing *X*. *maculatus* Jp 163 B females to *X*. *couchianus* males. The F_1_ animals sequenced are male that are 12 or 15 months old.

First generation backcross hybrid (BC_1_) *X*. *hellerii* × (*X*. *hellerii* × *X*. *couchianus*) used in this study were supplied by the XGSC. Specifically, *X*. *maculatus* female was artificially inseminated with sperm from a male *X*. *hellerii* to produce F_1_ hybrids. F_1_ hybrid males were then backcrossed to *X*. *hellerii* females to generate the BC_1_ animals. Skin and tumor samples were collected from *X*. *hellerii* × (*X*. *maculatus* × *X*. *hellerii*) BC_1_ animals that are 19–22 months old. One of the animals is a female but principle component analyses (S4 Fig) did not show segregation of the female data. Similarly, for another cross [i.e., *X*. *couchianus* × (*X*. *maculatus* × *X*. *couchianus*)], *X*. *maculatus* female was artificially inseminated with sperm from a male *X*. *couchianus* to produce F_1_ hybrids. F_1_ hybrid males were then backcrossed to *X*. *couchianus* females to generate the BC_1_ animals. Skin samples of 3 male and 5 female BC_1_ were sequenced. PCA showed no clustering of samples based on sex (S4 Fig).

Fifth generation backcross hybrids (BC_5_) were produced in an independent series of crosses from F_1_ hybrids originating from the reciprocal crosses where *X*. *maculatus* Jp163 A males were mated to *X*. *hellerii* (Lancetilla) females. The F_1_ hybrid females were then successively backcrossed to *X*. *hellerii* males to produce the fifth generation backcross hybrid (BC_5_) animals. All BC_5_ fish used in this study were from laboratory stocks maintained in the governmentally certified animal facilities of the Biocenter (University of Wuerzburg, Germany). Skin samples were collected from BC_5_ animals that are 19–22 months old.

All animals are kept in 13/11 hours light/dark cycle in the *Xiphophorus* Genetic Stock Center at 25°C, and are fed twice daily.

### Gene expression data and genotyping

All sequencing files were acquired from previous studies, including skin samples from 20 inbred *X*. *maculatus* fish sequenced in 6 batches, skin samples from 6 *X*. *couchianus* sequenced from 3 batches, skin samples from 6 *Sp-couch* sequenced in 3 batches, skin and melanoma samples from 14 *X*. *hellerii* × (*X*. *maculatus* × *X*. *hellerii*) BC_1_ from one single batch, and melanoma samples from 13 *X*. *hellerii* × (*X*. *maculatus* × *X*. *hellerii*) BC_5_ fish from one single batch [45–49], and skin samples from 8 *X*. *couchianus* × (*X*. *maculatus* × *X*. *couchianus*) BC_1_ fish from a single batch. All individual sequences were mapped to the *X*. *maculatus* genome (Ensembl Xmac.4.4.2) using Bowtie2 [50], and gene expression was subsequently quantified using a custom Perl script that count sequencing reads that mapped to gene models [33]. For technical details regarding gene expression assessment and genotyping, see [21,45].

### Human melanoma gene expression

A total of 473 gene expression profiles from human skin cutaneous melanoma (SKCM) were retrieved from The Cancer Genome Atlas (TCGA, tcga-data.nci.nih.gov) SKCM dataset through the TCGA data portal. A custom Perl script was used to combine the dataset and append a patient-specific sample name to the corresponding expression profiles.

### Assessment of gene expression coefficient of variation

Expression values of each gene were first normalized to the total read count of a sample (i.e., library size), followed with removal of batch effect using R/Bioconductor package edgeR “removeBatchEffect”. Gene expression Coefficient of Variation (CV) was calculated by dividing the standard deviation by the mean of gene expression at each locus among each population of *Xiphophorus* fish [12]. A density curve of CV was calculated for all genes per biological condition and plotted using R. A Kolmogorov-Smirnov test was used to test whether the CV values calculated from different populations were from the same data distribution. A p-value < 0.05 was used to determine if two CV distributions are statistically different from each other.

### Identification of genes exhibiting different expression variability

F-test (test of variation) was used to detect genes that showed different expression variability in parental species *X*. *maculatus* and *X*. *couchianus*, and interspecies hybrid between the two, by comparing the normalized gene expression values of parental species, to those of F_1_ interspecies hybrids. We selected genes that exhibited CV_F1_/CV_parental_ ≥ 4 or ≤ 0.25, with confidence level of 95% (p-value < 0.05)

### Genotype and gene expression association analyses

14 BC_1_ hybrid progeny were sequenced for gene expression profiling and allele specific gene expression analyses to estimate genotype. Not all genes were genotyped due to the statistic not being met when running our data analyses pipeline [21]. Genes that were able to be genotyped in all BC_1_ progeny, of which 7 were heterozygous and 7 were homozygous, were used for association analysis. A linear modeling between the genotype of each marker, and gene expression of each gene, was formed and association analysis performed using Analysis of Variance (ANOVA). For each genetic marker, multiple testing corrections were conducted using False Discovery Rate (FDR) correction. Markers (genetic variants) that had FDRs corrected p-value < 0.05 were determined to be statistically significantly affect gene expression.

### Differential gene expression analyses

For genes that were genotyped in BC_1_ animals, and genes of which the expression showed to be associated with local or remote genotype, the gene expression when they at homozygous state was compared to the expression when heterozygous. A t-test was used to assess whether gene expression of different genotypes were statistically significant. FDR correction was used to control the multiple testing. For a gene to be considered differentially expression, FDR adjusted p-value needs to be smaller than 0.05 (p-adjusted < 0.05).

### Allele specific gene expression

All transcript sequences of the *X*. *maculatus* were compared to those of *X*. *couchianus* using blast. Shared sequence sections of both alleles were preserved and compiled to form a Reciprocal Best Hits (RBH) reference transcriptome to represent transcriptome of interspecies hybrid between *X*. *maculatus* and *X*. *couchianus*. Sequencing reads from F_1_ interspecies hybrids were mapped to the RBH reference transcriptome using Bowtie2 “head-to-head” mode. Total gene expression was quantified using a custom perl script that counts the number of sequencing reads that mapped to a transcript regardless of allelic origin. Samtools was subsequently used to show sequencing reads that only mapped to polymorphic sites (i.e., SNP or In/Del) between parental allelic sequences and custom perl scripts were used to assign sequencing reads that cover the polymorphic site to one of the parental species. The sequencing depths of polymorphic sites per gene were used to calculate expression ratio of both parental alleles.

## Supporting information

S1 Fig Comparison of gene expression CV between F_1_ and inbred animals. Data set of inbred animals that have sample size matched to F_1_ animals was created by randomly select 6 inbred animals expression profiles, with expression CV calculated. This task was repeated 100 times to estimate whether the observation that F_1_ showed smaller CVs is due to the smaller sample size of F_1._ This test showed sample-size matched inbred animals showed CV distribution that is larger than F_1_ animals (a). Additionally, X-axis values where peak of density curve took place were used to calculate Δ Peak Position between inbred and F_1_ animals (Δ Peak Position = Peak Position inbred−Peak Position _F1_). (b) Histogram shows sample sized matched inbred animals exhibited CV density curve right shifted compared to F_1_ animals.
(PDF)

S2 Fig Allelic expression preference and coefficient of variation in interspecies F_1_. Gene expression of *X*. *maculatus* and *X*. *couchianus* parental species are compared to the *X*. *maculatus* × *X*. *couchianus* interspecies F_1_ to identify genes that show less variability in the hybrids (CV_F1_/CV_parental_ ≤ 0.25, p-value < 0.05; left: genes that show less variability in F_1_ interspecies hybrid than *X*. *maculatus*; right: genes that show less variability in F_1_ interspecies hybrid than *X*. *couchianus* parental species; Top: relative allelic expression CV in F_1_ vs. relative allelic expression in F_1_; Bottom: relative gene expression CV in parental species vs. relative gene expression in parental species). Allelic expression of the genes that show less variability in hybrid is assessed and allelic expression of both alleles are used to calculate parental allele contribution to total gene expression, and allelic expression variability. Each point represents a gene defined by X- and Y-values. X-value represents relative parental allelic expression: Positive values means *X*. *maculatus* allele dominants the expression, negative values means *X*. *couchianus* allele dominants the expression; Y-value represents the ratio of CV of *X*. *maculatus* and *X*. *couchianus* allelic expression. Genes with X-values > 2 are dominantly expressed by *X*. *maculatus* allele (i.e., *X*. *maculatus* accounts more than 80% of the expression). Genes with X-values < -2 are dominantly expressed by *X*. *couchianus* allele (i.e., *X*. *couchianus* accounts more than 80% of the expression). Gray dots represent allelic expression ratio and allelic expression CV ratio. Light blue dots represent parental species expression ratio and expression CV ratio. Black dot highlights the genes that are dominantly expressed by one of the parental allele in the F_1_ hybrid. Genes exhibiting less expression variability in F_1_ interspecies hybrid compare to *X*. *maculatus* (left) or *X*. *couchianus* (right) both show expression from parental alleles that showed less allelic expression variability.
(PDF)

S3 Fig Gene expression CV of genes that are regulated by cis- and trans-variant. Expression of 289 genes is associated with cis-variants and expression of 111 genes is associated with trans-variants. Among the 289 genes which expressions are associated to the genotypes of cis- or trans-variants, 216 are regulated by cis-variant only and 38 are regulated by trans-variant only. CV distribution is recalculated for the 216 and the 38 genes based on marker genes (i.e., variants) genotypes. Gene expression CV distribution of inbred parental (black line), F_1_ (gray line), all BC_1_ (light blue line) individuals.
(PDF)

S4 Fig Principle component analyses of backcross individual gene expression. 14 backcross hybrids between *X*. *maculatus* and *X*. *hellerii* (red) and 8 backcross hybrids between *X*. *maculatus* and *X*. *couchianus* (blue) formed sample sets that are sex-mixed. Principle component analyses was performed on these two datasets using the library size normalized expression counts. Male and female samples do not show separation along PC1 and PC2.
(PDF)

S1 Table Genes showing less expression variability in hybrid than parental species. (CSV)

S2 Table Genes showing stable expression variability in hybrid and parental species. (CSV)

S3 Table Genotype-gene expression association scores. (CSV)

S4 Table Differentially expressed genes between heterozygous and homozygous genes in BC_1_ progeny. (CSV)
