# Supplementary figures and images for: Gene expression variation and parental allele inheritance in a Xiphophorus interspecies hybridization model
Source: PLoS Genet. 2018 Dec 26;14(12):e1007875. doi: 10.1371/journal.pgen.1007875 (PMC6324826; doi:10.1371/journal.pgen.1007875)

(a)

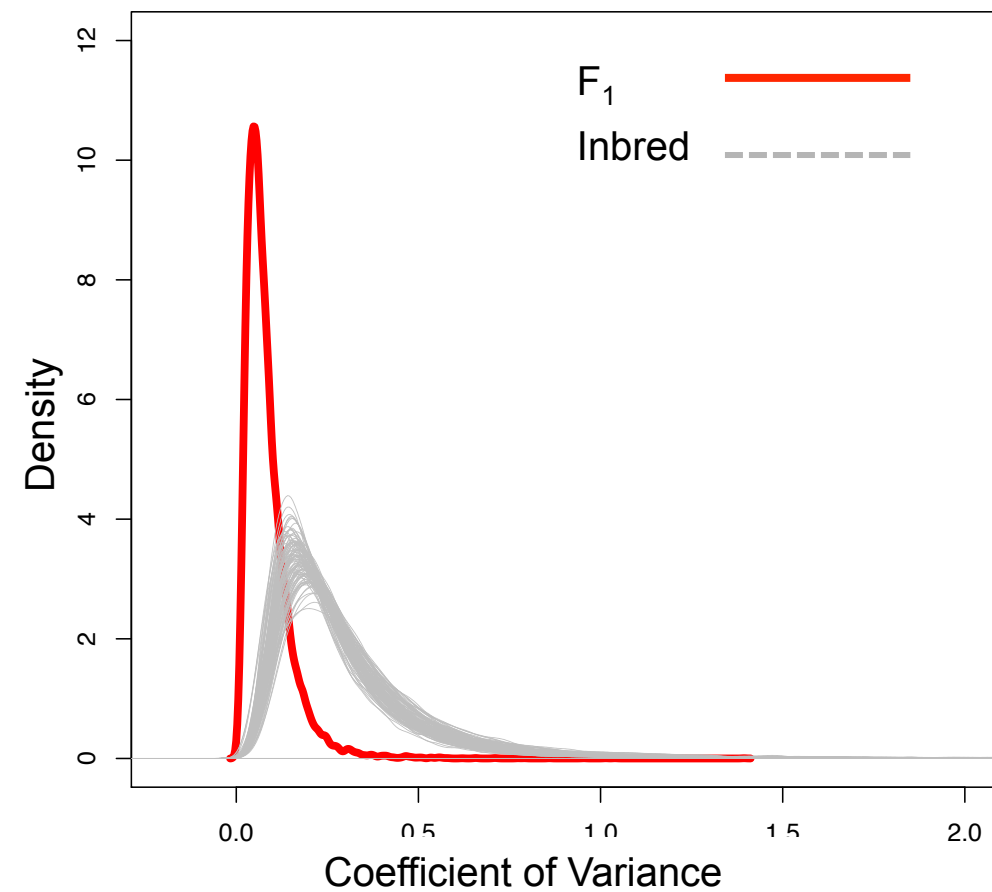

(b)

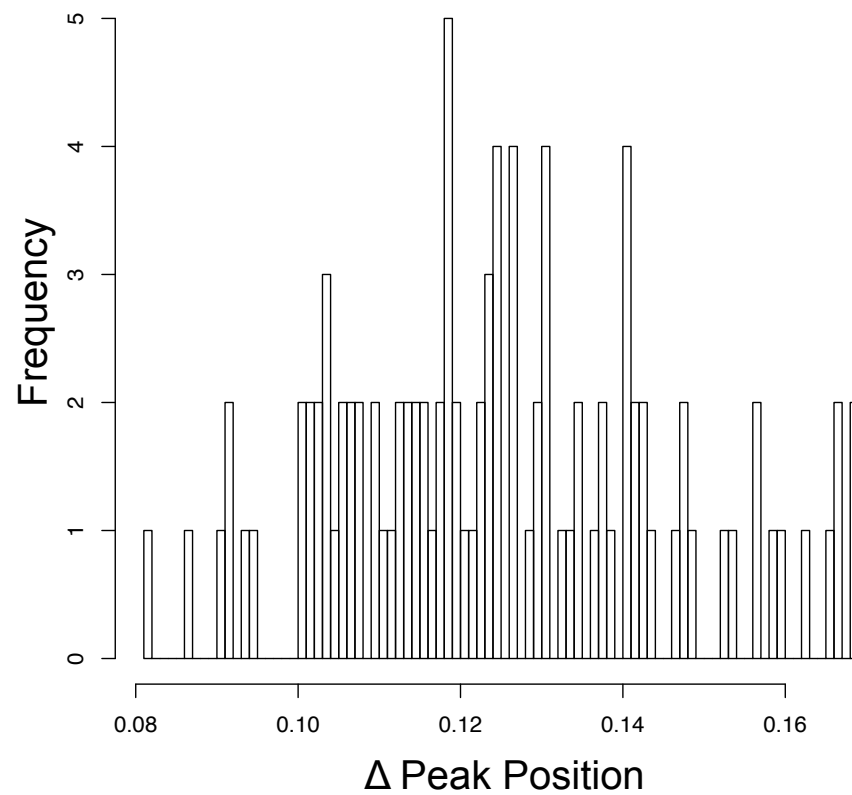

Supplement: S1 Fig — Data set of inbred animals that have sample size matched to F1 animals was created by randomly select 6 inbred animals expression profiles, with expression CV calculated. This task was repeated 100 times to estimate whether the observation that F1 showed smaller CVs is due to the smaller sample size of F1. This test showed sample-size matched inbred animals showed CV distribution that is larger than F1 animals (a). Additionally, X-axis values where peak of density curve took place were used to calculate Δ Peak Position between inbred and F1 animals (Δ Peak Position = Peak Position inbred−Peak Position F1). (b) Histogram shows sample sized matched inbred animals exhibited CV density curve right shifted compared to F1 animals. (PDF) [file pgen.1007875.s001.pdf]

Genes under cis-variants only

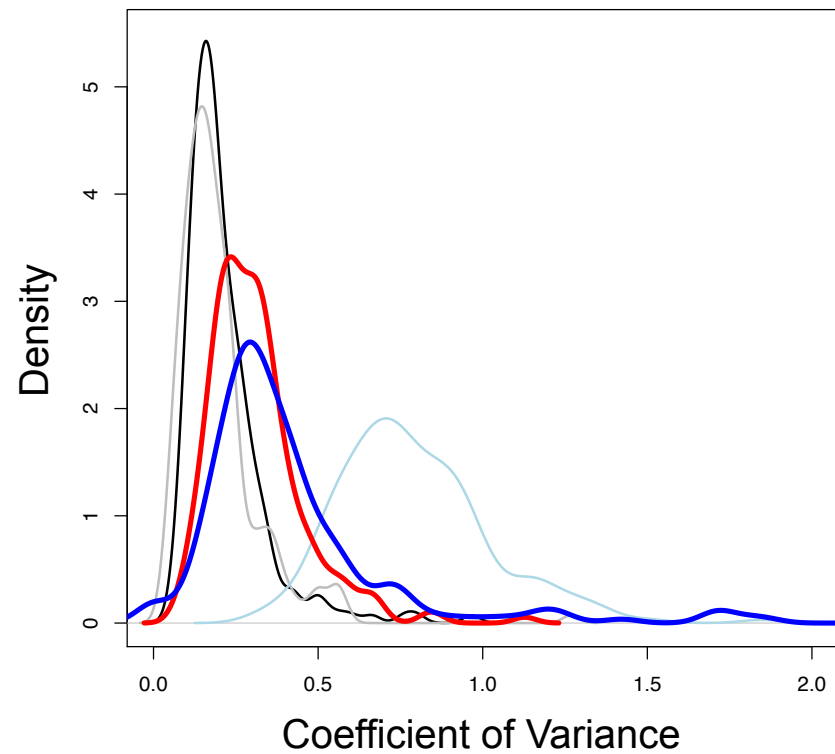

Genes under trans-variants only

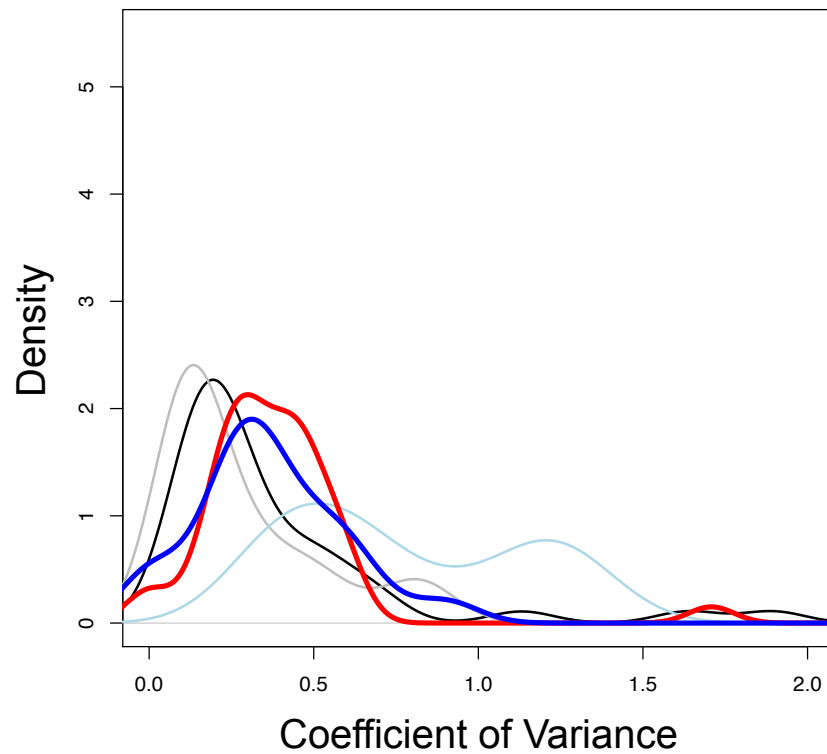

Supplement: S3 Fig — Expression of 289 genes is associated with cis-variants and expression of 111 genes is associated with trans-variants. Among the 289 genes which expressions are associated to the genotypes of cis- or trans-variants, 216 are regulated by cis-variant only and 38 are regulated by trans-variant only. CV distribution is recalculated for the 216 and the 38 genes based on marker genes (i.e., variants) genotypes. Gene expression CV distribution of inbred parental (black line), F1 (gray line), all BC1 (light blue line) individuals. (PDF) [file pgen.1007875.s003.pdf]

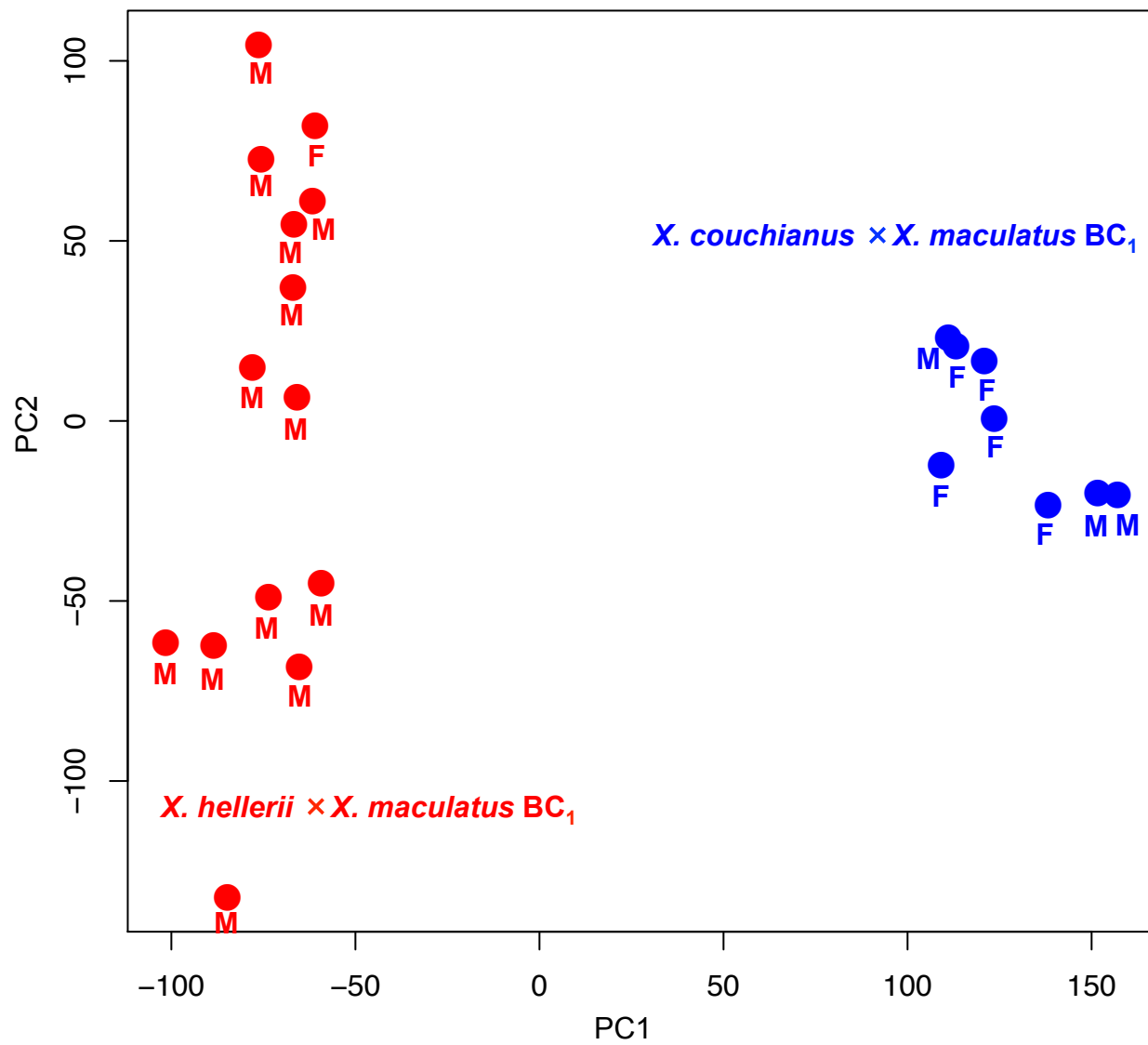

Supplement: S4 Fig — 14 backcross hybrids between X. maculatus and X. hellerii (red) and 8 backcross hybrids between X. maculatus and X. couchianus (blue) formed sample sets that are sex-mixed. Principle component analyses was performed on these two datasets using the library size normalized expression counts. Male and female samples do not show separation along PC1 and PC2. (PDF) [file pgen.1007875.s004.pdf]
