# Supplementary material for: Gene expression variation and parental allele inheritance in a Xiphophorus interspecies hybridization model
Source: PLoS Genet. 2018 Dec 26;14(12):e1007875. doi: 10.1371/journal.pgen.1007875 (PMC6324826; doi:10.1371/journal.pgen.1007875)
Supplement: S2 Fig — Gene expression of X. maculatus and X. couchianus parental species are compared to the X. maculatus × X. couchianus interspecies F1 to identify genes that show less variability in the hybrids (CVF1/CVparental ≤ 0.25, p-value < 0.05; left: genes that show less variability in F1 interspecies hybrid than X. maculatus; right: genes that show less variability in F1 interspecies hybrid than X. couchianus parental species; Top: relative allelic expression CV in F1 vs. relative allelic expression in F1; Bottom: relative gene expression CV in parental species vs. relative gene expression in parental species). Allelic expression of the genes that show less variability in hybrid is assessed and allelic expression of both alleles are used to calculate parental allele contribution to total gene expression, and allelic expression variability. Each point represents a gene defined by X- and Y-values. X-value represents relative parental allelic expression: Positive values means X. maculatus allele dominants the expression, negative values means X. couchianus allele dominants the expression; Y-value represents the ratio of CV of X. maculatus and X. couchianus allelic expression. Genes with X-values > 2 are dominantly expressed by X. maculatus allele (i.e., X. maculatus accounts more than 80% of the expression). Genes with X-values < -2 are dominantly expressed by X. couchianus allele (i.e., X. couchianus accounts more than 80% of the expression). Gray dots represent allelic expression ratio and allelic expression CV ratio. Light blue dots represent parental species expression ratio and expression CV ratio. Black dot highlights the genes that are dominantly expressed by one of the parental allele in the F1 hybrid. Genes exhibiting less expression variability in F1 interspecies hybrid compare to X. maculatus (left) or X. couchianus (right) both show expression from parental alleles that showed less allelic expression variability. (PDF) [file pgen.1007875.s002.pdf]

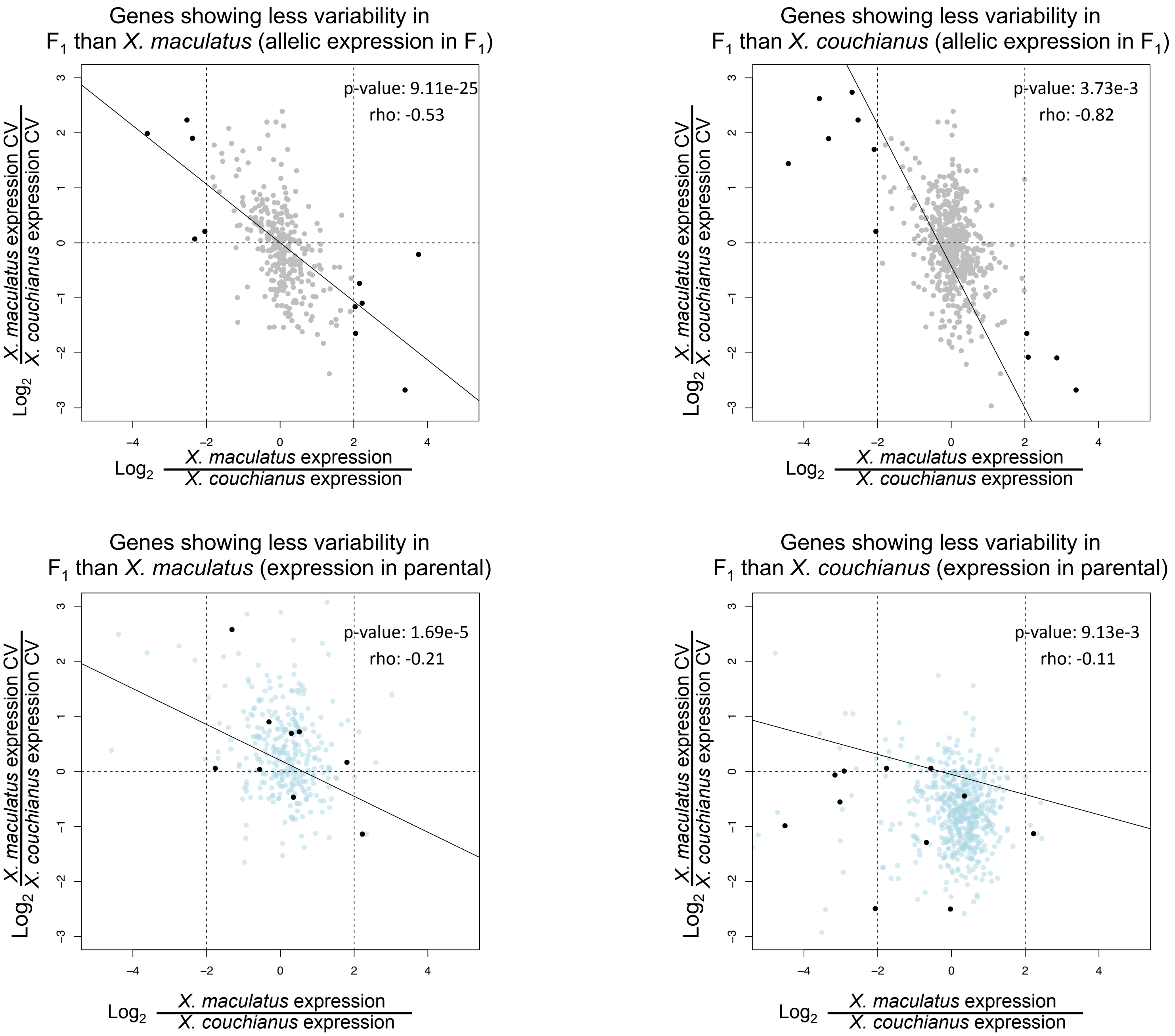
Genes showing less variability in F_1 than *X. maculatus* (allelic expression in F_1)
p-value: 9.11e-25
rho: -0.53
Genes showing less variability in F_1 than *X. couchianus* (allelic expression in F_1)
p-value: 3.73e-3
rho: -0.82
Genes showing less variability in F_1 than *X. maculatus* (expression in parental)
p-value: 1.69e-5
rho: -0.21
Genes showing less variability in F_1 than *X. couchianus* (expression in parental)
p-value: 9.13e-3
rho: -0.11
$\log_2 \frac{X.\ maculatus\ \text{expression CV}}{X.\ couchianus\ \text{expression CV}}$
$\log_2 \frac{X.\ maculatus\ \text{expression}}{X.\ couchianus\ \text{expression}}$
−4
−2
0
2
4
−3
−2
−1
0
1
2
3
